# LISTENING AND LEARNING TO FORM THE HBI ROAD MAP FOR INDIAN COUNTRY

**DOI:** 10.1093/geroni/igz038.1337

**Published:** 2019-11-08

**Authors:** Molly French, Michael Splaine, John Shean, Heidi Holt

**Affiliations:** 1 Alzheimer’s Association, Washington, District of Columbia, United States; 2 Splaine Consulting, Columbia, Maryland, United States; 3 Alzheimer’s Association, Washington DC, District of Columbia, United States; 4 CDC, Atlanta, Georgia, United States

## Abstract

American Indian and Alaska Native (AI/AN) communities are establishing new paths as more older adults develop Alzheimer’s and other dementias along with other co-morbidities. To offer a flexible framework of public health strategies that proactively address the growing issue of dementia among AI/ANs, Alzheimer’s Association and Centers for Disease Control and Prevention (CDC) developed the first-ever Healthy Brain Initiative Road Map for Indian Country. Partnering with International Association for Indigenous Aging supported Road Map development through virtual listening sessions and written comments from regional Native health experts, tribal aging service leaders, and tribal government officials. Many additional discussions, engagement of a cultural guide, and an additional partnership with National Indian Health Board further informed Road Map contents, graphic design, and marketing. Presenter will describe rationale for the process, themes from the consultations, and lessons learned by the Association and CDC that can apply to similar initiatives.

